# Expression and functional characterization of INPP4B in gallbladder cancer patients and gallbladder cancer cells

**DOI:** 10.1186/s12885-021-08143-6

**Published:** 2021-04-20

**Authors:** Youliang Wu, Delong Meng, Xin Xu, Junjun Bao, Yexiang You, Yanjun Sun, Yongxiang Li, Dengqun Sun

**Affiliations:** 1 Department of General Surgery, the First Affiliated Hospital of Anhui Medical University, Hefei, 230022 People’s Republic of China; 2 Department of Molecular Biology, University of Texas Southwestern Medical Center, 6000 Harry Hines Blvd, Dallas, TX 75390 USA; 3 Department of Gastroenterology, the First Affiliated Hospital of Anhui Medical University, Hefei, 230022 People’s Republic of China; 4 Department of General Surgery, the Armed Police Corps Hospital of Anhui, Hefei, 230041 People’s Republic of China

**Keywords:** Gallbladder cancer, INPP4B, Tumour suppressor gene, Oncogene, Prognostic biomarker

## Abstract

**Background:**

Inositol polyphosphate 4-phosphatase type II (INPP4B) is a negative regulator of the PI3K-Akt signalling pathway and plays a contradictory role in different types of cancers. However, the its biological role played by INPP4B in human gallbladder cancer (GBC) has not been elucidated. In this study, we investigated the expression, clinical significance and biological function of INPP4B in GBC patients and cell lines.

**Methods:**

The INPP4B protein expression levels in gallbladder cancer tissues and normal gallbladder tissues were detected by immunohistochemistry, and the clinical significance of INPP4B was analysed. Knockdown and overexpression of INPP4B in GBC-SD and SGC-996 cells followed by cell proliferation, clonogenic, apoptosis detection, scratch wound-healing and transwell assays were used to identify INPP4B function in vitro.

**Results:**

INPP4B was up-regulated in human GBC tissues compared with normal gallbladder tissues and was related to histopathological differentiation (*p* = 0.026). Here, we observed that INPP4B was highly expressed in high-moderately differentiated tumours compared with low-undifferentiated tumours (*p* = 0.022). Additionally, we found that INPP4B expression was not associated with overall survival of GBC patients (*p* = 0.071) and was not an independent prognostic factor. Furthermore, when we stratified the relationship between INPP4B expression and the prognosis of GBC based on histopathological differentiation, we found that INPP4B played a contradictory role in GBC progression depending on the degree of differentiation. In addition, INPP4B knockdown inhibited the proliferation, colony formation, migration and invasion in GBC cells, while INPP4B overexpression had the opposite effects in vitro, which indicates its role as an oncoprotein.

**Conclusions:**

These findings suggested that INPP4B may play a dual role in the prognosis of GBC depending on the degree of differentiation and that INPP4B might act as an oncogene in gallbladder cancer cells.

**Supplementary Information:**

The online version contains supplementary material available at 10.1186/s12885-021-08143-6.

## Background

Gallbladder cancer (GBC), which is the most common malignant tumour of the biliary system, has the characteristics of sex, geographical and racial biases [1, 2]. In recent years, although the diagnosis and treatment of GBC have substantially improved, the prognosis of GBC patients has not significantly improved, as the five-year survival rate is less than 5% and the average overall survival is only 6 months [3]. Therefore, elucidating the molecular mechanisms of GBC progression is critical for the development of new diagnostic and therapeutic strategies.

The PI3K-Akt signalling pathway plays an important role in tumour cell proliferation, differentiation, angiogenesis, invasion and metastasis [4, 5]. Hyper-activation of PI3K-Akt signalling has been shown to be the driving factor of tumour initiation and progression [6, 7]. The PI3K-Akt signalling pathway is considered a therapeutic target for a variety of malignant tumours, and many clinical trials have been performed to investigate the therapeutic effects of PI3K-Akt pathway inhibitors on human cancer [8]. Recently, as a phosphoinositide phosphatase, Inositol polyphosphate-4-phosphatase type II (INPP4B) has been demonstrated to be a negative regulator of PI3K-Akt signalling and to serve as a tumour suppressor gene (TSG) [9–12]. Low expression of INPP4B in several cancer types is associated with poor clinical outcomes [13–15]. Knockdown of INPP4B enhances the proliferation and migration of breast cancer, melanoma and prostate cancer cell lines, which, suggests its TSG role in these cancer cells [11, 15, 16]. However, some recent studies have reported that INPP4B is highly expressed in colon cancer, acute myeloid leukaemia and melanoma, where it serves as an oncogene and is a positively correlated with poor clinical outcomes [17–19]. Even within the same tumour, different researchers have reached varying conclusions about the role of INPP4B as an oncogene or TSG [16, 18–20]. The above-mentioned studies indicated that the expression and functional role of INPP4B are controversial in cancer, and seem to be tumour-specific. However, to date, the expression level, clinical prognostic value and biological function of INPP4B in GBC have not been studied. To promote the comprehensive understanding of the potential value of INPP4B in GBC, it will be useful to detect its expression level, evaluate its clinical prognostic significance and investigate its cell function in vitro.

In the present study, we detected the expression of INPP4B in GBC tissues and non-tumorous tissues by immunohistochemistry. We found that the expression of INPP4B in GBC tissues was significantly higher than that in non-tumorous tissues. INPP4B protein expression was decreased in low-undifferentiated GBC tissues but was increased in high-moderately differentiated tissues. In addition, high INPP4B expression was associated with a favourable prognosis in patients with high-moderately differentiated tumours, while poor prognosis was observed in patients with low-undifferentiated tumours. We further explored the biological function of INPP4B in GBC cells in vitro and found that INPP4B knockdown inhibited the proliferation, colony formation, migration and invasion in GBC-SD and SGC996 cells, while INPP4B overexpression had the opposite effects. These results suggested that INPP4B plays a critical role in GBC and may be a potential target for the treatment of GBC.

## Methods

### Patients and tissue specimens

In the present study, 127 GBC tissues and 47 non-tumorous tissues were collected from patients at the Armed Police Corps Hospital of Anhui (Hefei, China) for the analysis of INPP4B expression by immunohistochemistry. Detailed clinicopathological parameters are described in Table 1. Patients did not receive any anticancer treatment prior to surgery.

**Table 1:** Relationship between INPP4B expression and clinicopathological variables (*n* = 127)

Clinicopathological variables	Total	INPP4B *expression*		*p value*
		positive (58)	negative (69)	
Sex				0.862
Male	36	16	20	
Female	91	42	49	
Age (y)				0.984
< 68	59	27	32	
≥ 68	68	31	37	
Tumor size (cm)				0.116
< 2	67	35	32	
≥ 2	60	23	37	
Differentiation				**0.026**
High/moderate	81	43	38	
Low/undifferentiated	46	15	31	
Depth of invasion				0.754
T1/T2	39	17	22	
T3/T4	88	41	47	
Lymph node metastasis				0.405
Yes	51	21	30	
No	76	37	39	
TNM				0.735
I/II	83	37	46	
III/IV	44	21	23	
Gallstones				0.321
Yes	84	41	43	
No	43	17	26	
AFP (ug/L)				0.999
< 20	122	56	66	
≥ 20	5	2	3	
CEA (ng/ML)				0.771
< 5	100	45	55	
≥ 5	27	13	14	
CA199 (U/ML)				0.735
< 37	83	37	46	
≥ 37	44	21	23	

*TNM* tumor-node-metastasis, *AFP* alpha fetoprotein, *CEA* carcino-embryonic antigen, *CA199* carbohydrate antigen 199

*p* < 0.05 was defined statistically significant

### RNA preparation, reverse transcription and real-time qPCR

Total RNA was extracted from GBC cells using TRIzol Reagent (Invitrogen). Reverse transcription (RT) was performed using ReverTra Ace qPCR RT Master Mix (Toyobo) according to the manufacturer’s instructions to obtain first-strand cDNA. To amplify the cDNAs, qPCR was then performed with SYBR-Green mix (Toyobo, Japan) on an ABI Prism 7900 HT Sequence Detection System (Applied Biosystems, USA). The PCR primers used for amplification were as follows: INPP4B, 5′-ACGCAGGAAAGTCAGGCTAA-3′ (forward), 5′-TGCCAGGTAACACC ATTTCTT-3′ (reverse); GAPDH served as an endogenous control, 5′-ATCAAGAAGGTGGTGAAGCAGG-3′ (forward), 5′-CGTCAAAGGTGGAGGAGTGG- 3′ (reverse). The relative expression levels of INPP4B were calculated using the 2^−ΔΔCT^ method. Each sample was measured in triplicate.

### Immunohistochemistry (IHC)

Immunohistochemistry (IHC) was used to detect the expression of INPP4B in 4-μm-thick formalin-fixed paraffin-embedded human GBC tissues and non-tumorous tissues. IHC and staining evaluation were performed as previously reported [21, 22]. The tissue sections were incubated with a primary monoclonal antibody against INPP4B (Abcam, ab81269, 1:50), while negative controls were incubated with normal rabbit IgG (Beyotime Institute of Biotechnology, A7016). The immunohistochemical staining results were evaluated by two independent pathologists who were not informed of the patients’ clinical information. According to the intracellular immunoreactive staining of GBC and normal mucosal cells, the staining percentage of positive mucosal cells was graded on a scale of 0 to 4 (0 points, no cells stained; 1 point, 1–25% positive cells; 2 points, 26–50% positive cells; 3 points, 51–75% positive cells; 4 points, 76–100% positive cells). The staining intensity of mucosal cells was graded on a scale of 0 to 3 (0 points, negative; 1 point, weak intensity; 2 points, moderate intensity; 3 points, strong intensity). The immunoreactivity score (IRS) is obtained by multiplying the two parameters (staining intensity×staining percentage). The IRS ranged from 0 to12. We defined an IRS = 0 as no expression, 0 < IRS < 4 as weak expression, 4 ≤ IRS ≤ 8 as moderate expression and 8 < IRS ≤ 12 as strong expression. We combined the no expression and weak expression groups into the negative group (INPP4B^−^, IRS < 4), while the moderate expression and strong expression groups were combined into the positive group (INPP4B^+^, IRS ≥ 4).

### Cell culture and lentivirus infection

The GBC cell lines GBC-SD and SGC996 were obtained from Genechem (Shanghai, China) and were cultured in RPMI1640 medium supplemented with 10% FBS, penicillin (100 U/ml) and streptomycin (100 μg/ml) in a humidified incubator at 37 °C with 5% CO2. In order to establish GBC-SD and SGC996 cells with stable INPP4B overexpression, the two cell lines were infected with the INPP4B overexpression lentivirus (GV492-INPP4B, OEINPP4B) or control lentivirus (GV492, OECtrl, purchased from Genechem, Shanghai, China). In addition, to establish stable INPP4B knockdown GBC-SD and SGC996 cells, the two cell lines were infected with lentiviral shRNA against INPP4B (GV248-shINPP4B) or control lentivirus (GV248-shCtrl, purchased from Genechem, Shanghai, China). The target sequence of shINPP4B was 5′-CCATCTGAGTATCCCATCTAT-3′. The overexpression and knockdown efficiency of the target genes were detected by q-PCR and western blot.

### Cell proliferation and clonogenic assays

Cell proliferation and clonogenic assays were employed to measure the role of INPP4B in GBC cell viability and proliferation ability. The detailed cell proliferation assay procedure was performed as previously described [23, 24]. Briefly, different lentiviruses (OEINPP4B, OECtrl; shINPP4B, shCtrl) were used to infect GBC cells (GBC-SD and SGC-996), which were seeded into 96-well plates (approximately 2000 cells/well) in sextuplicate, after which cell viability was determined by MTT assay (Genview, JT343) at each 24 h interval according to the manufacturer’s instructions. The number of viable cells was determined by measuring the absorbance at OD490 nm using a Universal Microplate Reader (BioTek Instruments, Inc.). Each experiment was performed three times. The detailed colony formation assay procedure was performed as previously described [25, 26]. Briefly, different lentivirus-infected GBC cells were seeded into 6-well plates (approximately 800 cells/well) and the medium was replaced every 3 days. After 2 weeks, the colonies were fixed in 4% polyoxymethylene, stained with Giemsa, and counted using an inverted microscope. Each assay was repeated three times.

### Apoptosis assay

The detailed apoptosis analysis protocol has been described in previous studies [27]. Briefly, according to the manufacturer’s instructions, the apoptosis level of different lentivirus-infected GBC cells was analysed using the Annexin V-APC apoptosis detection kit (eBioscience, 88–8007). In our study, we only used the Annexin V-APC kit to test the apoptosis levels; the virus we used expresses GFP protein, and the green fluorescence represents virus-infected cells. Q1 refers to the cells infected with virus but not stained by Annexin V-APC, which are the cells infected with virus that were not apoptotic; Q2 and Q3 refer to the cells infected with virus but also stained by Annexin V-APC, which are apoptotic cells; Q4 represents cells not infected by virus. Therefore, the percentage of apoptotic cells was calculated by the addition of Q2 and Q3. The apoptosis rate was analysed by flow cytometry (FACSCalibur; BD Biosciences).

### Scratch wound-healing and Transwell assays

Cell migration and invasion abilities were assessed by scratch wound-healing and Transwell assays, which were performed as previously described [28, 29]. Briefly, for the scratch wound-healing assay, different lentivirus-infected GBC cells were seeded in 96-well plates (approximately 5 × 10^5^ cells/well). The next day, after the cells had adhered to the bottom, linear wounds were created by scratching the centre of the cell monolayer. At different time points, wounds images were obtained using a Celigo instrument, and the migration area of different lentivirus-infected GBC cells was analysed by this software. Briefly, for the Transwell assay, different lentivirus-infected GBC cells in 0.2 mL serum-free medium (approximately 1 × 10^5^ cells/well) were added to the upper chamber of 24-well plates containing 8-μm pores (Corning, 3422), and 0.6 mL DMEM (Corning, 10–013-CVR) containing 30% FBS (Ausbian, A11–102) was added to the lower chamber. The plate was incubated for 24 h after which non-invading cells were removed with cotton swabs; the remaining cells were fixed in 4% paraformaldehyde for 30 min, stained with 0.5% crystal violet, and counted under a microscope at 200× magnification.

### Statistical analysis

SPSS 16.0 software (SPSS, Inc.) and GraphPad Prism 5 were used for the statistical analysis. A Pearson χ2 test was used to assess the relationship between INPP4B expression and clinicopathologic characteristics. Data are expressed as the mean ± SEM. Student’s t test was used to evaluate the statistical significance of two groups. Survival analysis was performed using the Kaplan-Meier method and the Cox proportional hazards regression model. *p* < 0.05 was considered statistically significant. (*p < 0.05, **p < 0.01, ***p < 0.001 and #*p* < 0.0001, ns, not significant).

## Results

### INPP4B is increased in GBC tissues and plays a prognostic role that differs according to the degree of GBC tumour tissue differentiation

**Fig. 1:**
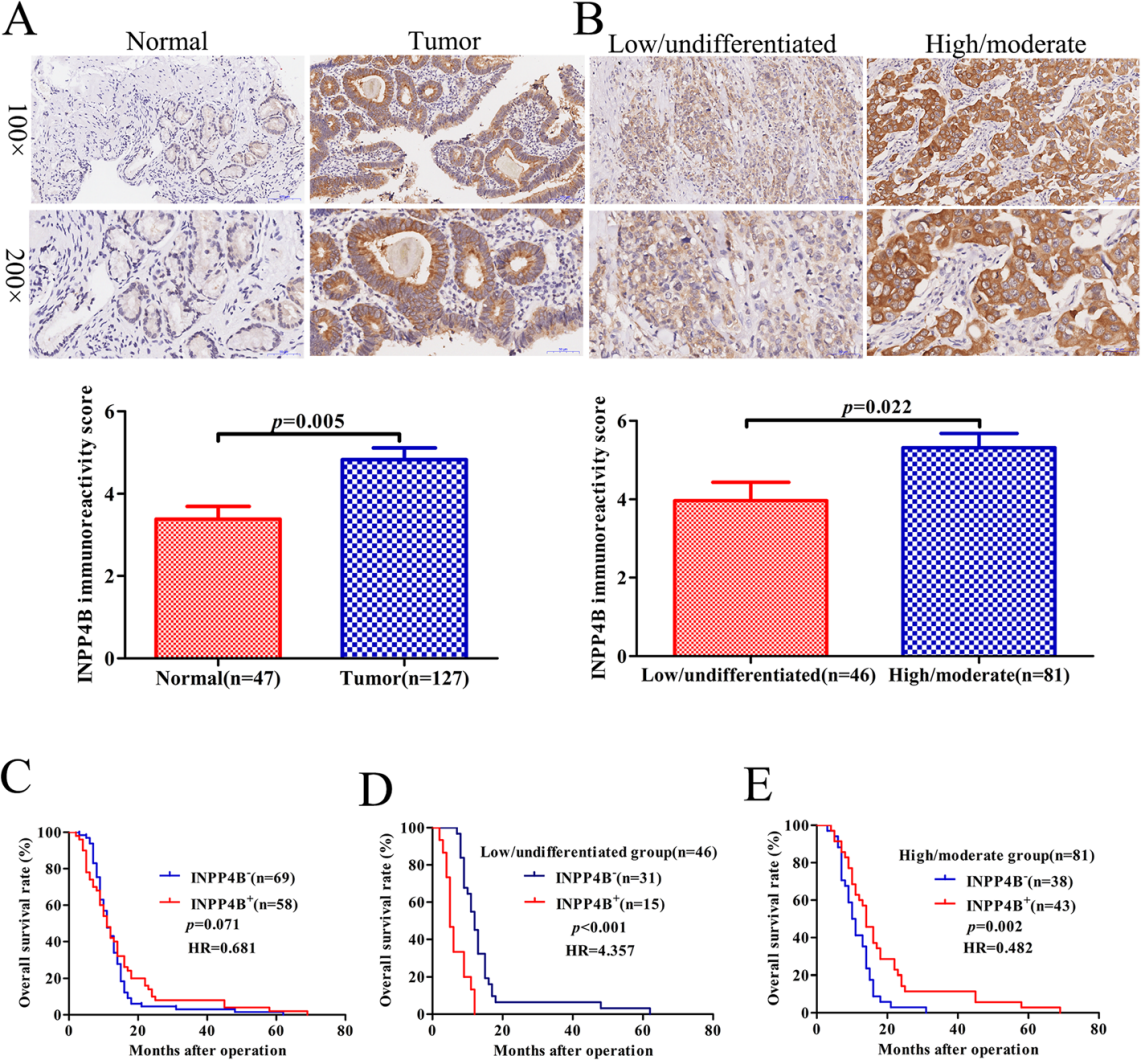
Protein expression levels and prognostic significance of INPP4B in GBC clinical samples. **a** Representative images and immunoreactivity scores of INPP4B in tumour tissues and normal tissues. **b** Representative images and immunoreactivity scores of INPP4B in low-undifferentiated and high-moderately differentiated tumour tissues. **c** The overall survival of GBC patients with INPP4B^−^ and INPP4B^+^ tumours. **d** The overall survival of GBC patients in the low-undifferentiated group with INPP4B^−^ and INPP4B^+^ tumours. **e** The overall survival of GBC patients in the high-moderate differentiated group with INPP4B^−^ and INPP4B^+^ tumours

INPP4B protein expression in tumours is still controversial and has not been studied in GBC tissues. We analysed INPP4B protein expression in 127 GBC tissue samples and 47 non-tumorous tissues by immunohistochemistry. As a result, INPP4B staining was mainly observed in the cell cytoplasm, and the positivity rate was significantly higher in GBC tissues (58/127) than in non-tumorous tissues (11/47, *p* = 0.008). Representative images are shown in Fig. 1a. The results reveal that INPP4B is highly expressed in GBC and is mainly located in the cytoplasm.

In addition, when we further elucidated the correlation between INPP4B expression and GBC clinicopathological parameters, we found that INPP4B expression was closely associated with histopathological differentiation (*p* = 0.026, Table 1). The IRS of high-moderately differentiated tumours (5.324 ± 0.353) was higher than that of low-undifferentiated tumours (3.967 ± 0.468, *p* = 0.022, Fig. 1b). These data suggest that INPP4B may play an important role in GBC histopathological differentiation.

Based on the above findings, we speculated that INPP4B might be related to GBC prognosis. However, a Kaplan-Meier analysis and log-rank test revealed that INPP4B expression was not associated with overall survival (OS) of GBC patients (*p* = 0.071, Fig. 1c). A multivariate analysis showed that INPP4B was not an independent prognostic factor (Table 2). However, when we stratified the relationship between INPP4B expression and prognosis of GBC patients in terms of histopathological differentiation, we obtained some interesting findings. In the low-undifferentiated group, we found that GBC patients with INPP4B^+^ showed worse prognosis (mean 6.5 months) than that of patients with INPP4B^−^ (mean 14.6 months, HR = 4.357, *p* < 0.001, Fig. 1d); while in high-moderate differentiation group, we found that GBC patients with INPP4B^+^ showed better prognosis (mean 22.4 months) than that of patients with INPP4B^−^ (mean 12.6 months, HR = 0.482, *p* = 0.002, Fig. 1e). These results indicate that INPP4B has a contradictory role as a prognostic factor of GBC progression according to histopathological differentiation.

**Table 2:** Univariate and multivariate analysis of the correlation between clinicopathological parameters and prognostic significance of GBC patients (n = 127)

Variables	Univariate analysis	*p* value	Multivariate analysis	*p* value
	HR(95%CI)		HR(95%CI)	
Sex (male vs. female)	1.316 (0.860–2.013)	0.205		NA
Age (y) (< 68 vs. ≥68)	1.265 (0.871–1.837)	0.217		NA
Tumor diameter (cm) (< 2 vs. ≥2)	1.424 (0.983–2.063)	0.061		NA
Differentiation (low/undifferentiated vs. high/moderate)	0.566 (0.387–0.827)	**0.003**	0.667 (0.428–1.041)	0.075
Depth of invision (T1/TI vs. T3/T4)	1.683 (1.101–2.572)	**0.016**	1.359 (0.850–2.172)	0.200
Lymph node metastasis (no vs. yes)	1.829 (1.249–2.676)	**0.002**	2.537 (1.123–5.730)	**0.025**
TNM stages (I/II vs. III/IV)	1.653 (1.123–2.433)	**0.011**	0.616 (0.263–1.441)	0.264
Gallstones	1.026 (0.698–1.508)	0.896		NA
AFP (< 20 vs. ≥20)	2.107 (0.853–5.207)	0.106		NA
CEA (< 5 vs. ≥5)	1.236 (0.790–1.932)	0.353		NA
CA199 (< 37 vs. ≥37)	1.198 (0.816–1.758)	0.356		NA
INPP4B expression (positive vs. negative)	0.681 (0.467–0.993)	0.086		NA

Variables with *p* values more than 0.05 in the univariate models were not adapted (NA) in the multivariate analysis. *p* < 0.05 was defined statistically significant and was given in bold

*CI* confidence interval, *HR* Hazard ratio

### INPP4B regulates GBC cell proliferation in vitro

**Fig. 2:**
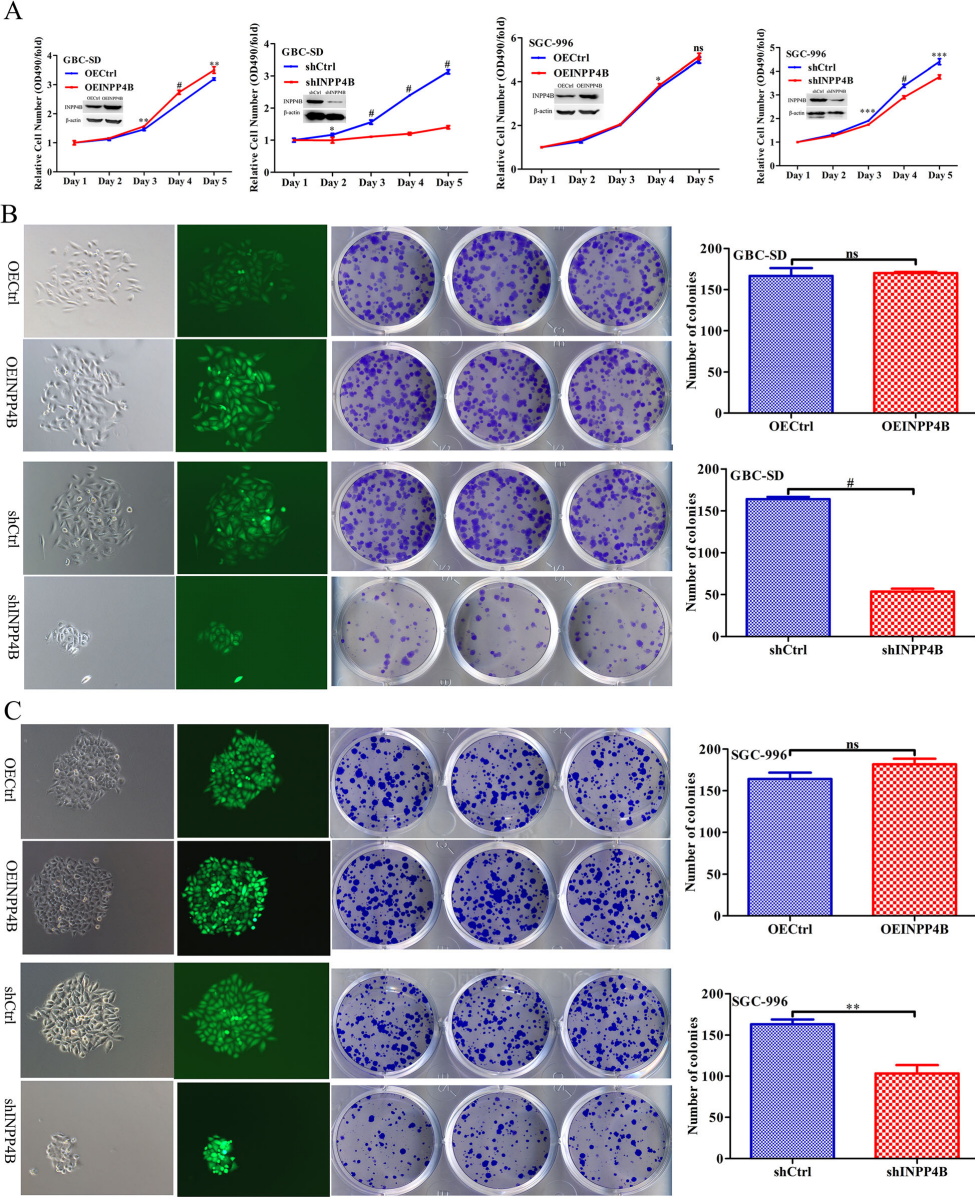
INPP4B regulates GBC cell growth in vitro. **a** Proliferation curve for GBC-SD and SGC996 cells in which INPP4B was overexpressed or knocked down and the negative control. **b**, **c** Colony formation of GBC-SD and SGC996 cells in which INPP4B was overexpressed or knocked down and the negative control. *, *p* < 0.05; **, *p* < 0.01; ***, *p* < 0.001; #, *p* < 0.0001; ns, not significant

Given the high expression of INPP4B in GBC tissue and its correlation with the clinical prognosis of GBC patients, we inferred that INPP4B might regulate GBC cell growth. To confirm our hypothesis, we selected GBC-SD and SGC996 cells for in vitro assay. GBC-SD and SGC996 control cells and cells with stable INPP4B overexpression and knockdown were established by infection with different lentiviruses (Fig. 2a). Subsequently, we examined the effects of INPP4B on the growth and proliferation of GBC-SD and SGC996 cells using MTT and clonogenic assays. As shown in Fig. 2a, b and c, blocking the endogenous INPP4B expression led to reductions in cell proliferation and and colony formation of 55.19% (*p* < 0.001) and 67.68% (*p* < 0.001), respectively, in GBC-SD cells and 14.47% (*p* < 0.001) and 36.81% (*p* = 0.007), respectively, in SGC996 cells, whereas overexpression of INPP4B weakly promoted the proliferation and colony formation of these cells. In summary, our findings suggest that inhibition of endogenous INPP4B expression has a greater effect on the proliferation of GBC cells than overexpression.

### INPP4B regulates GBC cell apoptosis in vitro

**Fig. 3:**
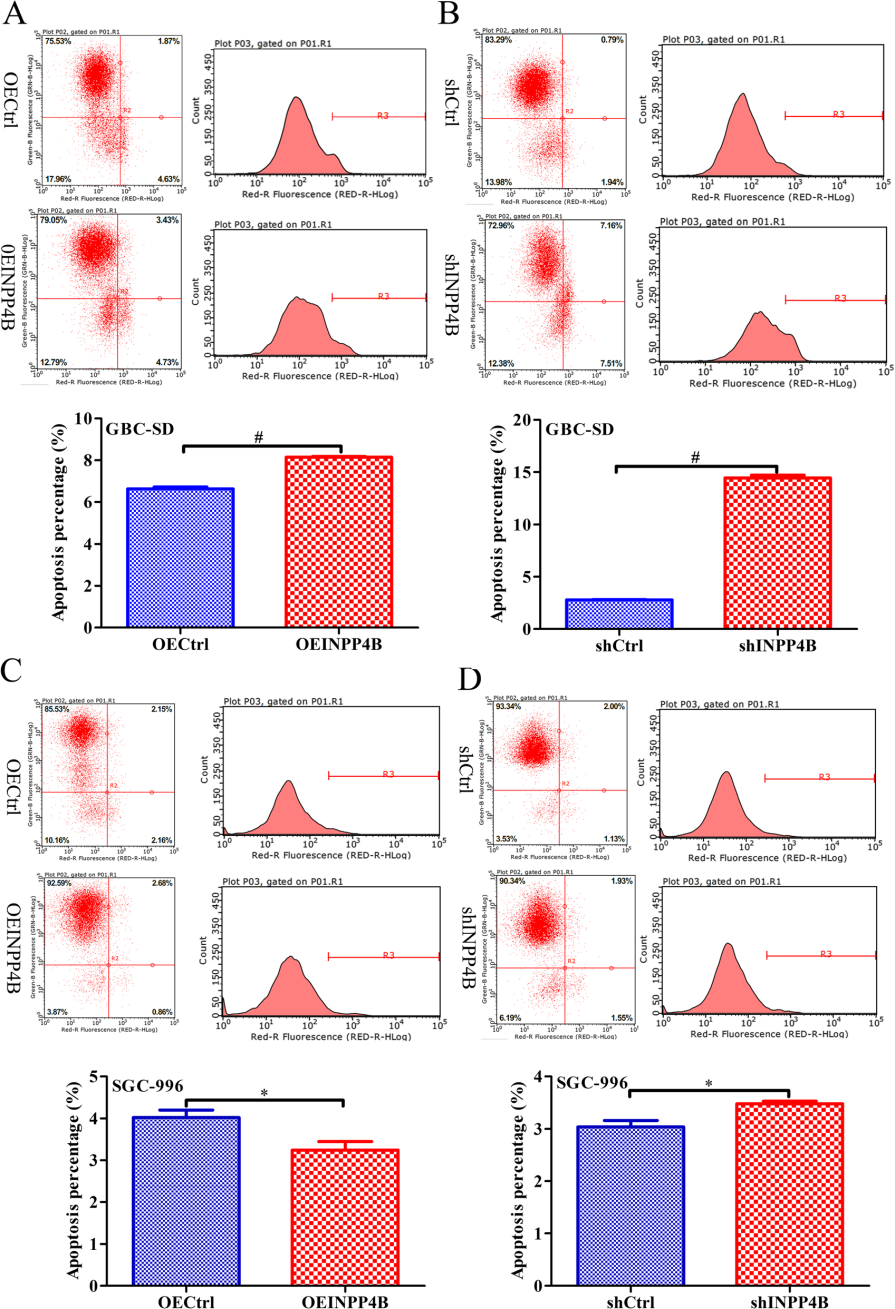
INPP4B controls GBC cell apoptosis in vitro. **a**, **b** Both INPP4B overexpression and knockdown all significantly promote GBC-SD cell apoptosis. **c** INPP4B overexpression significantly inhibits SGC996 cell apoptosis. **d** INPP4B knockdown significantly induces SGC996 cell apoptosis. *, *p* < 0.05; #, *p* < 0.0001

Previous studies suggested that INPP4B is involved in tumour cell apoptosis [15, 30]. The apoptosis levels in GBC-SD and SGC-996 cells infected with different lentiviruses were analysed by flow cytometry. INPP4B overexpression and knockdown increased the apoptosis rate in 1.51% (*p* < 0.001) and 11.66% (*p* < 0.001) of GBC-SD cell, respectively; INPP4B overexpression reduced the apoptosis rate in 0.79% (*p* = 0.041), while INPP4B knockdown increased the apoptosis rate in 0.45% (*p* = 0.025) of SGC-996, respectively. Our results showed that both INPP4B overexpression and knockdown significantly increased the apoptosis rate of GBC-SD cell (Fig. 3a and b). However, in SGC-996 cell, INPP4B overexpression markedly reduced the apoptosis rate, while INPP4B knockdown significantly increased the apoptosis rate (Fig. 3c and d). Our results suggest that INPP4B regulates apoptosis of GBC cells, but that the regulatory effects are distinct in different cell lines.

### INPP4B promotes GBC cell migration and invasion in vitro

**Fig. 4:**
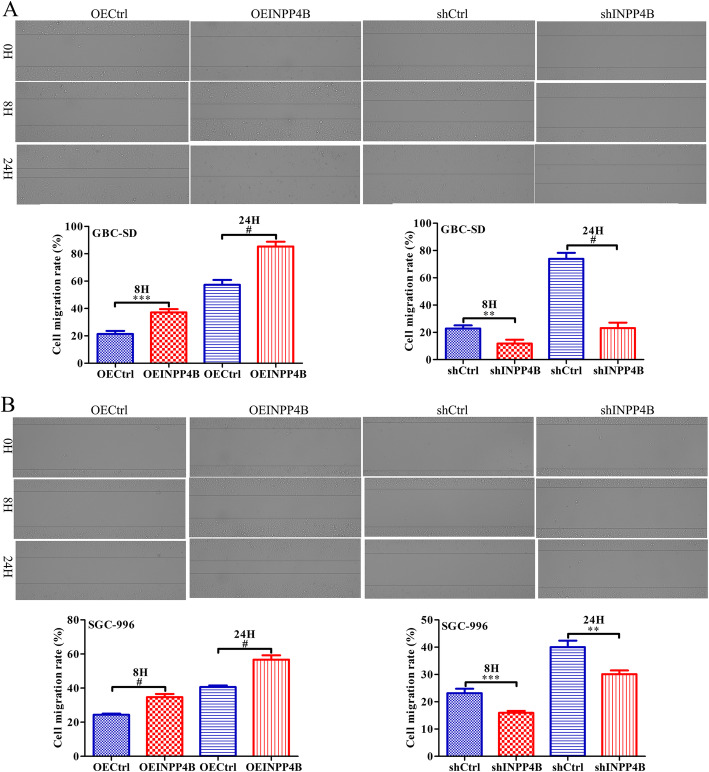
INPP4B regulates GBC cell migration ability in vitro. The migration of GBC-SD and SGC-996 cells after infection with different lentiviruses was detected by scratch wound-healing assays. **a** INPP4B overexpression significantly promotes GBC-SD cell migration, while INPP4B knockdown significantly inhibits GBC-SD cell migration. **b** INPP4B overexpression significantly promotes SGC996 cell migration, while INPP4B knockdown significantly inhibits SGC996 migration. **, *p* < 0.01; ***, *p* < 0.001; #, *p* < 0.0001; ns, not significant

**Fig. 5:**
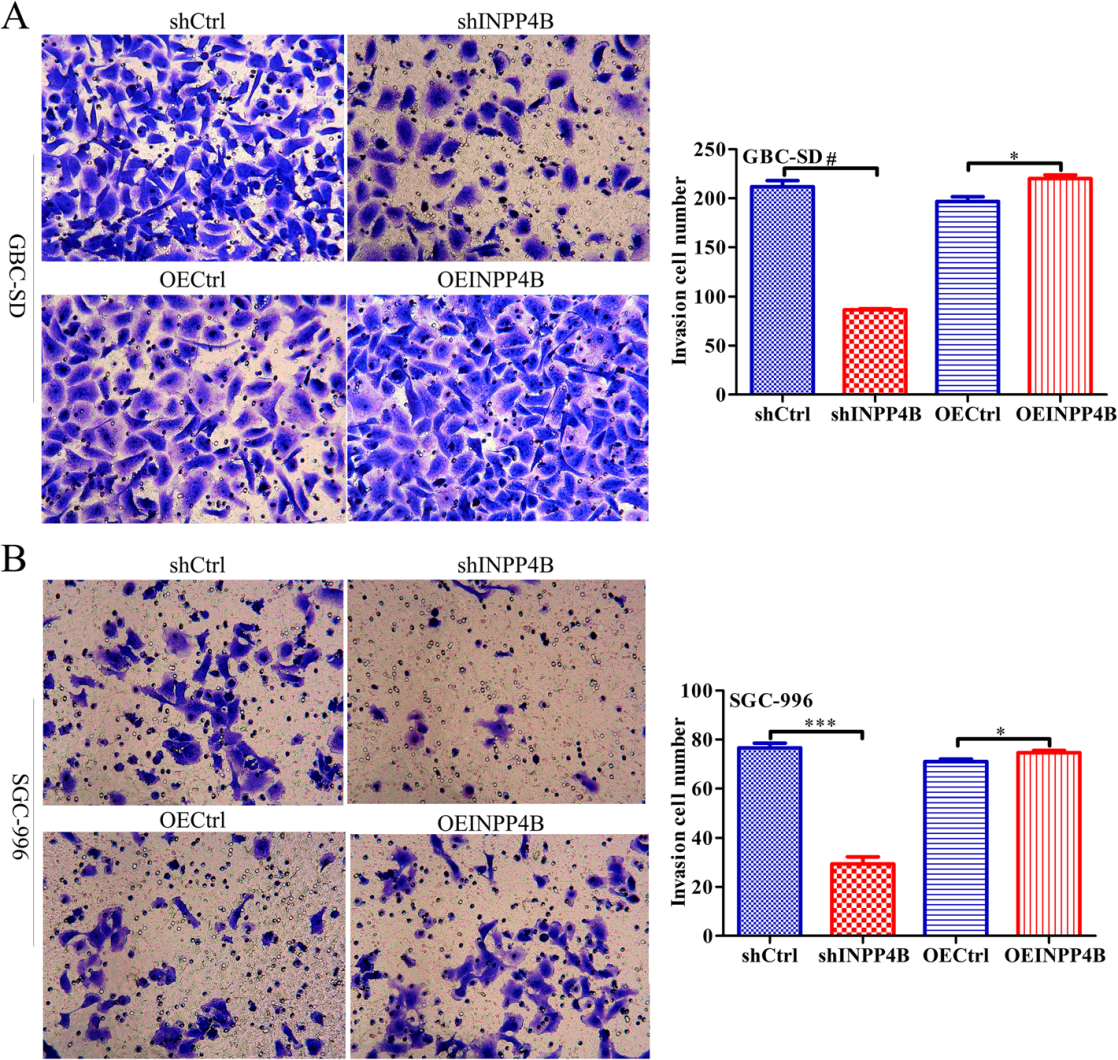
INPP4B regulates GBC cell invasion ability in vitro. The invasiveness of GBC-SDl and SGC-996 cells after infection with different lentiviruses was detected by Transwell assays. **a** INPP4B overexpression significantly promotes GBC-SD cell invasion, while INPP4B knockdown significantly inhibits GBC-SD cell invasion. **b** INPP4B overexpression significantly promotes SGC996 cell invasion, while INPP4B knockdown significantly inhibits SGC996 invasion. *, *p* < 0.05; ***, *p* < 0.001; #, *p* < 0.0001

Scratch wound-healing and Transwell assays were used to further investigate the effect of INPP4B on the migration and invasion ability of GBC cells. A scratch wound-healing assay confirmed that INPP4B overexpression increased the migration rate of GBC-SD cells by 15.74% (*p* < 0.001) and 28.02% (*p* < 0.0001) at 8 h and 24 h, respectively, and increased the migration rate of SGC996 cells by 10.33% (*p* < 0.001) and 16.11% (*p* < 0.001) at 8 h and 24 h, respectively, while INPP4B knockdown had the opposite effect on the migration ability of these cells (Fig. 4a and b). Consistent with these results, Transwell assays demonstrated that INPP4B overexpression increased the average cell invasion of GBC-SD (220 vs 197, *p* = 0.019) and SGC996 (75 vs 71, *p* = 0.026), while INPP4B knockdown had the opposite effect on their invasion ability (Fig. 5a and b). Taken together, these data suggest that INPP4B promotes GBC cell migration and invasion ability in vitro.

## Discussion

As a phosphoinositide phosphatase, INPP4B has been reported to be expressed at low levels, and to plays a tumour suppressive role in human prostate cancer, breast cancer and ovarian cancer by negatively regulating PI3K-Akt signaling [10, 11, 13]. More recently, some unexpected findings have indicated that INPP4B is significantly upregulated, and plays an oncogenic role in AML, melanoma and colon cancer by activating SGK3, and that it is associated with patient prognosis [17–19, 31]. To date, previous studies on the role of INPP4B as a tumour suppressor gene and oncogene, even in the same tumour, have been controversial. Guo et al. reported that INPP4B is frequently upregulated in human colon cancer tissues and cell lines where it promotes tumorigenesis [18]. Sung et al. and Ma et al. reported that INPP4B is down-regulated and has a tumour suppressor role in colorectal tumours [20]. However, the expression and clinical significance of INPP4B in human GBC and its biological function in GBC cells have not been studied.

In the present study, we first revealed that INPP4B is highly expressed in GBC tissues compared with non-tumour tissues and is associated with the prognosis of GBC patients in different histopathological differentiation groups. When we investigated the correlation between INPP4B expression and clinicopathological parameters and clinical prognosis, we obtained some interesting findings. Table 1 and Fig. 1b reveal that INPP4B protein expression was associated with histopathological differentiation and that INPP4B expression was higher in high-moderately differentiated tissues than in low-undifferentiated tissues. When we did not stratify the relationship between INPP4B expression and GBC prognosis, survival analysis and Cox regression analysis showed that INPP4B was not associated with overall survival in GBC patients and was not an independent prognostic factor (shown in Table 2 and Fig. 1c). When we stratified the relationship between INPP4B expression and GBC prognosis according to differentiation grade, we found that GBC patients with high INPP4B expression had a better prognosis in high-moderate differentiation group, but a worse prognosis in low-undifferentiated group, which demonstrates its contradictory role. These results highlight the dual role of INPP4B in terms of GBC prognosis in tumours with different histopathological differentiation grades. These findings seem to be consistent with the report by Yang et al., which revealed that INPP4B plays a tumour suppressor role in non-metastatic colorectal cancer stem-like cells (CR-CSLCs) and plays an oncogene role in metastatic CR-CSLCs according to different mechanisms, although INPP4B is weakly expressed in non-metastatic CR-CSLCs and highly expressed in metastatic CR-CSLCs [32]. These results suggest that even in the same tumour, INPP4B tends to promote tumour progression in more malignant tissues and cells, while it inhibits tumour progression in relatively less malignant tissues and cells, regardless of its expression. This may be one of the reasons why various researchers have reached different conclusions about tumour progression in INPP4B. Next, we studied the function of INPP4B in GBC cells.

Previous studies on the role of INPP4B in tumour cells have shown that INPP4B functions as a tumour suppressor gene or oncogene in different types of tumour cells. INPP4B acts as a tumour suppressor gene in breast cancer cells and prostate cancer cells. Its knockdown can promote the proliferation and motility of breast cancer cells [10], and its overexpression can inhibit the migration, invasion and angiogenesis of prostate cancer cells [33]. In contrast, INPP4B acts as an oncogene in AML cells and colon cancer cells. INPP4B promotes AML cell growth [17], and INPP4B silencing inhibits colon cancer cell proliferation and retards colon cancer xenograft growth [18]. In our study, we explored the function of INPP4B in two GBC cell lines (GBC-SD, SGC996) using proliferation, colony formation, apoptosis, migration and invasion assays. Our result showed that knockdown of INPP4B in GBC-SD and SGC996 cells significantly suppressed proliferation, colony formation, migration and invasion ability; by contrast, overexpression of INPP4B in these two GBC cell lines notably increased migration and invasion ability but weakly promoted cell proliferation and colony formation to different degrees. These findings suggest that INPP4B, mainly regarded as a tumor suppressor gene, may play a dual role acting as a potential oncoprotein in GBC. When we analysed the effect of INPP4B on apoptosis of these two GBC cell lines, we observed some interesting phenomena. INPP4B overexpression in SGC996 cells significantly reduced the apoptosis rate, while INPP4B knockdown notably increased the apoptosis rate. However, in GBC-SD cells, it was confusing that both overexpression and knockdown of INPP4B all increased the apoptosis rate. This may have been due to the multiple complex INPP4B -related carcinogenic signalling pathways that are activated in different cells, which needs further study. In addition, this also reflects the heterogeneity of the two cell lines used in our study, and thus, more biological function tests should be performed in more cell lines to validate the results and to provide evidence for targeted therapy in different patients. In conclusion, our study suggests that INPP4B may play an important role in the modulation of GBC cell progression.

In summary, we first found that INPP4B is upregulated in GBC tissues by immunohistochemistry and that this protein plays a contradictory prognostic role in the progression of GBC according to histopathological differentiation. We found that GBC patients in the high-moderate differentiation group whose tumours had high expression of INPP4B had a better prognosis, whereas those in the low-undifferentiated group had a worse prognosis. In vitro cell experiments further confirmed that INPP4B may act as an oncogene in GBC cells. We found that INPP4B knockdown could inhibit proliferation and colony formation, decrease cell migration and invasion, and increase the apoptosis rate of GBC-SD and SGC996 cells; in contrast, INPP4B overexpression had the opposite effect on these biological behaviours in GBC-SD and SGC996 cells, except that it also increased the apoptosis rate of GBC-SD cells. These findings suggest that INPP4B may play an important role in the pathogenesis and development of GBC. However, this study has some limitations that cannot be ignored. First, the number of enrolled patients in this study was relatively small, and more cases should be used to more accurately assess INPP4B expression and its relationship with prognosis in GBC. Second, only two gallbladder cancer cell lines were used. More gallbladder cancer cell lines should be used to increase the accuracy of the experimental results. In addition, the reason why both INPP4B overexpression and INPP4B knockdown can increase the apoptosis rate of GBC-SD cells is still not completely understood. Moreover, we only studied the function of INPP4B in GBC cells in vitro, and further study is needed in vivo. Finally, this study only shows that INPP4B plays an important role in the development of GBC from clinical significance to cell function studies. We should further explore the regulatory mechanism by which INPP4B causes GBC progression, which can help us develop new methods for the clinical treatment of GBC. This is what we plan to study in future work.

## Conclusions

In conclusion, our study is the first to assess the clinical significance and function of INPP4B in GBC. Our results demonstrate that INPP4B is highly expressed in GBC tissues and is significantly associated with poor overall survival in low-undifferentiated GBC patients, and with better overall survival in high-moderately differentiated GBC patients. In addition, we found that INPP4B can promote wound healing, migration, invasion and proliferation in vitro, which suggests that INPP4B may be a potential therapeutic target in GBC patients. Further research will focus on the mechanisms underlying the potential for targeting INPP4B in GBC treatment.

## Supplementary Information

**Additional file 1.**

## Data Availability

The datasets used and/or analysed in the current study are available from the corresponding author on reasonable request.

## Abbreviations

AML: Acute myeloid leukaemia
GBC: Gallbladder cancer
IHC: Immunohistochemistry
INPP4B: Inositol polyphosphate 4-phosphatase type II
IRS: Immunoreactivity score
OS: Overall survival
TSG: Tumour suppressor gene
