# Supplementary material for: Expression and functional characterization of INPP4B in gallbladder cancer patients and gallbladder cancer cells
Source: BMC Cancer. 2021 Apr 20;21:433. doi: 10.1186/s12885-021-08143-6 (PMC8056679; doi:10.1186/s12885-021-08143-6)
Supplement: Supplementary file 1 — Additional file 1. [file 12885_2021_8143_MOESM1_ESM.doc]

Figure S1. Knockdown efficiency of INPP4B in GBC-SD and SGC-996 cells. The first two lanes represent the knockdown efficiency of INPP4B in GBC-SD cell, and the last two lanes represent the knockdown efficiency of INPP4B in SGC-996 cell.





Figure S2. β-actin as the internal control. The first two lanes represent the internal control of the knockdown efficiency of INPP4B in GBC-SD cell, and the last two lanes represent the internal control of the knockdown efficiency of INPP4B in SGC-996 cell.





Figure S3. Overexpression efficiency of INPP4B in GBC-SD and SGC-996 cells. The first two lanes represent the overexpression efficiency of INPP4B in GBC-SD cell, and the last two lanes represent the overexpression efficiency of INPP4B in SGC-996 cell.





Figure S4. β-actin as the internal control. The first two lanes represent the internal control of the overexpression efficiency of INPP4B in GBC-SD cell, and the last two lanes represent the internal control of the overexpression efficiency of INPP4B in SGC-996 cell.
